# Physical Proximity With Social Support Regulates Vigilance to Threat: Evidence From Startle Reactivity During Emotional Stress Induction

**DOI:** 10.1111/psyp.70259

**Published:** 2026-02-10

**Authors:** Antonio Maffei, Fiorella Del Popolo Cristaldi, Alessia Tecchio, Terry D. Blumenthal, Paola Sessa

**Affiliations:** ^1^ Department of Developmental Psychology and Socialization University of Padova Padova Italy; ^2^ Padova Neuroscience Center University of Padova Padova Italy; ^3^ Department of General Psychology University of Padova Padova Italy; ^4^ Department of Psychology Wake Forest University Winston‐Salem North Carolina USA

**Keywords:** emotion regulation, social baseline theory, social support, startle reflex, stress buffering, trier social stress test

## Abstract

Access to social support during emotional stress is one of the most important factors for the successful regulation of stress‐induced psychophysiological activation, and is predictive of improved health and well‐being. In this research we wanted to deepen our understanding of this buffering effect, focusing on the modulation of the startle reflex during a standardized stress induction as a function of the proximity with social resources as well as the relationship type with them. Seventy participants were exposed to the Trier Social Stress Test (TSST) in one of three possible conditions: Alone, Together with their romantic partner, or Together with a stranger. Startle reactivity to a series of acoustic probes presented during the task was measured along with self‐reported levels of anxiety. Results indicate that, independently of the social manipulation, stress induction is associated with a strong inhibition of the startle reflex. Furthermore, we found that access to social resources buffers this startle inhibition, showing that being together with others when facing a stressor regulates threat vigilance. We interpret these findings through the lens of the Social Baseline Theory, suggesting that startle dynamically tracks the load sharing process by which proximity with social resources optimizes the physiological as well as cognitive regulation of behavior in a threatening environment.

## Abstract 2

Being close to others has a beneficial impact on stress‐induced physiological reactivity. Tracking the modulation of the startle reflex during stress, we showed that the stress buffering selectively modulates the reflex, suggesting that simple social presence supports a more efficient allocation of attentional resources under threat. This work provides novel evidence of how social environments dynamically shape core defensive responses, offering new insights on emotional co‐regulation.

## 1 Introduction

Social presence and social support are powerful instrumental sources of emotional regulation, playing a critical role in mitigating the psychophysiological impact of negative emotions. Decades of longitudinal epidemiological research show that individuals who experience social isolation are characterized by higher levels of the hypothalamic–pituitary–adrenal axis (HPA) activity (Cacioppo et al. 2014), increased levels of systemic inflammation markers (Quadt et al. 2020), an increased risk of developing cardiovascular diseases, and an overall increased morbidity and mortality risk (Holt‐Lunstad et al. 2015; Wang et al. 2023). On the other hand, individuals who live in a supportive social environment characterized by positive and emotionally fulfilling relationships tend to have improved health and a lower risk of mortality and morbidity. Despite the substantial evidence linking social support to positive health outcomes, the psychobiological mechanisms underlying this connection remain unclear. Many authors suggest that a critical determinant of the relationship between social support and health is the buffering role played by the presence of a supportive social network when an individual has to face stressful situations (Brown et al. 2018; Ditzen and Heinrichs 2014; Uchino et al. 2012). This stress‐buffering effect is widely studied using laboratory‐based stress induction paradigms where participants face a stressful task either alone or benefiting from the presence and/or support of another person. A large number of studies employing this kind of paradigm reported a lower engagement of the stress response system, both in terms of hypothalamic–pituitary and sympatho‐adreno‐medullary axis, in participants facing a stressful task together with someone else rather than alone (Ditzen et al. 2008; Overall et al. 2022; Schwerdtfeger and Schlagert 2011). Furthermore, they report clear evidence of a more pronounced physiological buffering effect when support comes from a close person (e.g., a romantic partner) than a stranger (Ditzen et al. 2008; Maffei et al. 2025; Overall et al. 2022).

The Social Baseline Theory (SBT; Beckes and Coan 2011; Beckes and Sbarra 2022; Coan and Sbarra 2015) provides the theoretical framework to explain these findings. The main tenet of SBT is that the human brain expects the availability of social resources to cope with environmental demands, reflecting our species' adaptation to a fundamentally social ecological niche. This means that the metabolic cost of dealing with stressful and demanding tasks without access to social resources (e.g., facing a threat alone) is high because our brain (and body) did not evolve to operate optimally in isolation.

SBT identifies two interrelated mechanisms—load sharing and risk distribution—to explain why proximity with social resources (and support) is so important (Coan and Sbarra 2015). These mechanisms suggest that when a threat arises in the environment, the load as well as the effort to deal with it can be distributed among members of a social group, thus optimizing the metabolic and behavioral resources invested by individuals. This idea is supported by experimental findings showing how brain regions typically involved in threat reaction and emotional regulation are less (rather than more) engaged when social resources are available (Beckes et al. 2013; Coan et al. 2006, 2017). Interestingly, in this context, access to social resources does not necessarily mean receiving direct instrumental support (e.g., hand‐holding, affective touch). Instead, even simpler forms of social proximity—such as co‐presence or mental activation—can effectively reduce psychophysiological reactivity to threats (Bourassa et al. 2019; Maffei et al. 2025).

In this study, we wanted to understand if and how these mechanisms impact startle reactivity and habituation during stress. The startle reflex is a fast, involuntary motor response to a sudden and intense stimulus, such as a loud noise, which serves the purpose of protecting the body from potential threats (Zheng and Schmid 2023). This reflex, which is one of the most conserved defensive reactions across species, is mediated by a neural circuit primarily involving the brainstem, specifically the pontine reticular formation, and can be extrinsically modulated by other regions such as the amygdala (Davis et al. 2003). In humans, it is typically studied as the contraction of the muscle *orbicularis oculi* in response to a loud noise (> 90 dB), and it has been proven to be modulated by several factors, including emotional state, attention, and habituation. Startle magnitude is amplified when the startling sounds (i.e., startle probes) are paired with an emotionally negative stimulus, a phenomenon called affective startle potentiation. The startle reflex is also modulated by attention, because its magnitude is reduced when attention is, voluntarily or involuntarily, diverted away from the startle probes (Blumenthal and Flaten 1994; Deuter et al. 2012; Haerich 1994; Hamm et al. 1997; Lang et al. 1990). Finally, the progressive reduction of startle magnitude to repeated probes (i.e., habituation) is a sensitive index of information processing. Altogether, these features make the startle reflex an ideal tool to study the interaction between emotional and attentional processes.

The startle reflex has been extensively used to study attentional and emotional interaction in response to different kinds of experimental threats, such as predictable/unpredictable (NPU‐threat task) electrical shocks or aversive images (Dichter et al. 2002; Lissek et al. 2007; Schmitz and Grillon 2012), but surprisingly, very few studies investigated how ecological stressors with a socio‐evaluative component impact startle reactivity. In one study, Deuter and colleagues found that startle magnitude during the cold pressor test, a common physical stress task, is reduced (Deuter et al. 2012). Similarly, De La Casa and colleagues found that startle magnitude is reduced during a highly challenging and stressful mental task (De la Casa et al. 2016). The observed reduction contradicts the motivational priming hypothesis, which predicts that aversive contexts should potentiate (increase) startle responses, as negative emotional states typically amplify defensive reflexes. Instead, the startle inhibition suggests that during a stressful/challenging task, startle is primarily modulated by attentional mechanisms. Cuthbert et al. (1996) showed that motivational priming of startle by picture content required highly arousing pictures, and that at lower levels of rated picture arousal, both pleasant and unpleasant pictures resulted in attenuated startle, as would be expected by attention being diverted from the acoustic (startle stimulus) to the visual (picture) modality. Indeed, in the context of stress induction, the intense attentional demands of coping with stressors may reduce the processing resources available for the startle‐eliciting probes, thereby dampening the reflex. Thus, the more sensitive feature of stress induction is startle inhibition, rather than its potentiation.

In this study, we aimed to test whether this dynamical adjustment of startle reactivity during stress induction can be further modulated by the availability or unavailability of social resources, as well as by the level of emotional closeness with them. According to SBT, proximity to social resources would lead to a more efficient distribution of cognitive resources during stress induction, especially when the social resources are represented by a close person, such as a romantic partner. Thus, in this study, we wanted to understand whether startle reactivity would be a sensitive measure of this process.

To accomplish this goal, we designed a study where participants underwent a standardized psychosocial stress induction, the Trier Social Stress Test (TSST, Allen et al. 2016; Kirschbaum et al. 1993), in one of three possible conditions: (i) facing the stressor alone, (ii) facing the stressor together with their partner, present in the same room with them, or (iii) facing the stressor together with an unfamiliar person, present in the same room with them. Based on previous findings and on the SBT predictions, we advanced several hypotheses. First, we predicted that, independently of the manipulation of the social context, startle magnitude would decrease from baseline to stress induction, replicating previous evidence for attention‐mediated startle inhibition under stress (Hypothesis 1).

Second, we hypothesized that the presence of another person would influence startle reactivity during the task. Specifically, we should expect that startle would decrease less in participants in the two Social conditions, compared to those assigned to the Alone condition (Hypothesis 2). Such a pattern would indicate that social support optimizes resource allocation during stress. Alternatively, if social support plays no role, we should expect a similar trend of startle inhibition in the three groups.

Third, we predicted that, if proximity to others shapes startle reactivity under stress, this latter effect would be more pronounced in the group of participants doing the task together with their partner compared to those doing the task together with a stranger (Hypothesis 3).

## 2 Method

### 2.1 Participants

The sample size was estimated a priori using G*Power, which suggested a minimum sample of 45 participants for detecting a small‐to‐medium effect size (*f* = 0.2) in the within‐between interaction with a power of 80%. Based on this power analysis, we conducted a convenience sampling from a university student pool, recruiting 70 healthy young adult females (Mean age = 23.5 years, SD = 1.5 years) to participate in the research in exchange for course credits. They were all Italian citizens and identified as white, heterosexual females. Participants were divided into three groups according to the following criteria. One group comprised participants who were involved in a heterosexual romantic relationship for at least 6 months and were invited to participate in the experiment together with their male romantic partner (Partner present condition, *N* = 32, mean relationship length = 9 months). The second and third groups comprised heterosexual participants who were invited to participate in the experiment together with either a male stranger (Stranger present condition, *N* = 23) or alone (Alone condition, *N* = 15).

For the Partner present and Stranger present conditions, the female participant was assigned the role of the *target* of the stress induction procedure, while the male participant was assigned the role of the *observer*. We focused only on females as stress targets to minimize the confounding effects of gender differences in affective reactivity (Barnett et al. 2021; Maffei et al. 2015; Maffei and Angrilli 2019). Participants had no history of neurological, cardiovascular, or psychiatric diseases, and were not taking any medication that could influence startle reactivity. They were asked to refrain from taking caffeine and smoking in the 2 h preceding the experimental session. All procedures were carried out in accordance with the principles outlined in the Declaration of Helsinki for human research. This study's design and its analysis were not pre‐registered.

### 2.2 Stress Induction Procedure

Psychosocial stress was induced in the *target* participant with the public speaking task of the Trier Social Stress Test (TSST, Allen et al. 2016; Kirschbaum et al. 1993), which is the gold standard for experimental induction of a robust psychophysiological stress response in the laboratory.

**FIGURE 1:**
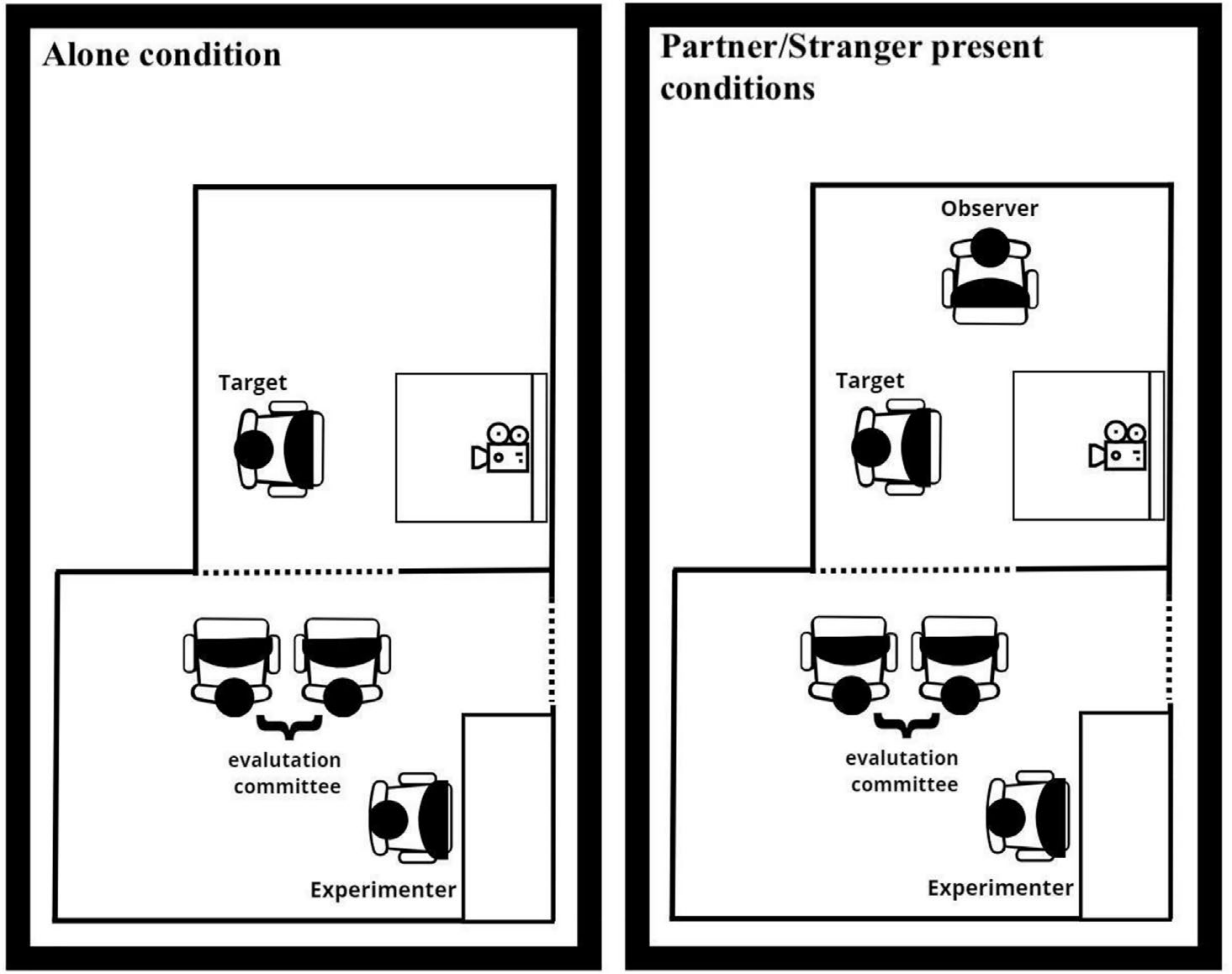
Schematic depiction of the experimental setting. The left panel shows the setting for the Alone condition. The right panel shows the setting for the Partner and Stranger present conditions.

Once arrived, participants were informed about the study procedures, asked to provide their signed informed consent to participate in the study, and given a 15‐min acquaintance with the laboratory environment before the experiment began. Then, they were led to a dimly lit sound‐attenuated room and asked to sit comfortably. Participants in the Alone group were alone in the experimental cabin, while participants in the Partner present or Stranger present conditions were asked to sit comfortably next to each other (Figure 1).

Specifically, Target participants assigned to the Partner group were invited to the laboratory session and asked to bring their partner for participating in the experiment with them. Upon arrival, they were introduced to their different roles and to the task. Target and Observer participants assigned to the Stranger group were individually invited to the laboratory for the experimental session. Upon arrival, the experimenters verified that the two participants did not know each other, and then they were introduced to their different roles and to the task.

The stress induction task comprised four distinct phases: (1) baseline, (2) stress anticipation, (3) acute stress, and (4) recovery, each lasting 5 min. The stressor consisted of first preparing (anticipation phase) and then delivering (acute stress phase) a mock job interview in front of an interviewing panel. To further increase social pressure and emotional stress, *target* participants were told that the interview would be videotaped for further analysis by public speaking experts.

The *observer* participant was instead instructed to rest and observe the target participant throughout the entire experimental session, without explicitly interacting with them. These instructions prevented observers from becoming an additional stressor. We made it explicit that observers were not judges or evaluators, thereby limiting their role to simple physical presence rather than an additional source of socio‐evaluative threat.

Since the two participants (target and observer) were in the same room, the instructions were given to both at the same moment.

### 2.3 Startle Elicitation

The startle reflex was elicited by presenting to the *target* participants a series of 10 white noise probes (50 ms duration, instantaneous rise, 95 dB amplitude, binaural presentation) with an ISI varying randomly between 15 and 45 s during each phase of the TSST. Since initial startle responses are typically exaggerated, we included a habituation phase before the beginning of the TSST, consisting in the presentation of five startle probes, during which participants were familiarized with the startle‐eliciting sounds.

### 2.4 Psychophysiological Data Collection and Preprocessing

Startle reactivity was measured from the contraction of the *orbicularis oculi* muscle using two Ag/AgCl electrodes attached below the target participant's left eye. The EMG signal was collected and amplified through a BrainAmp amplifier (BrainProducts, Munich, Germany) with a sampling rate of 2500 Hz. The raw EMG signal was offline preprocessed using custom MATLAB scripts. The preprocessing comprised the following steps: (i) band‐pass filtering (21–340 Hz), (ii) full‐wave rectification, (iii) smoothing with a 50 ms moving average filter, (iv) segmentation around probe onset (−50 ms, +500 ms), (v) baseline correction, and (vi) visual inspection of preprocessed epochs to detect and discard those contaminated by artifacts (e.g., eye‐blinks, electrical noise). The average percentage of good trials per condition were: Baseline = 94.3%, Anticipation = 85.4%, Speech = 66.3%, Recovery = 84.6%. Finally, startle reactivity was quantified from artifact‐free epochs as the maximum EMG peak in the 20–200 ms time window following probe onset. Absolute startle magnitudes were standardized *within‐subject* in T scores, following international guidelines on recording and analysis of startle reactivity (Blumenthal et al. 2005). To evaluate potential differences in the average number of trials, we ran a linear mixed‐effect model (LMM) which confirmed that the average number of artifact‐free trials was comparable across the three groups for each phase of the TSST (*F*_(6,202)_ = 1.32, *p* = 0.24), reducing the risk of biasing our estimates.

### 2.5 Subjective Measures of Stress Reactivity

Changes in participants' emotional state induced by the stress induction procedure were measured at the end of each phase of the task, using the state form of the State–Trait Anxiety Inventory (STAI‐Y1). The STAI is one of the most used self‐report instruments for measuring state anxiety levels, consisting of 20 items in which participants express how they feel at the moment using a 4‐point Likert scale (Spielberger and Sydeman 1994).

Once the experiment was finished, we asked the participants in the Partner and Stranger groups to rate on a 0–10 scale how much the presence of the other person made them feel (a) supported and (b) uncomfortable.

### 2.6 Statistical Analysis

LMMs were used to analyze the dependent variables considered in this study, namely, startle magnitude and STAI. For each model, we included as fixed predictors the independent variables Time (4 levels: baseline, anticipation, speech, recovery), Group (3 levels: partner, stranger, alone), and their interaction. The non‐independence of the observations was modeled by including the random effect for the participant. An F‐test with Satterthwaite correction for degrees of freedom was used to test the significance of the fixed predictors, and the size of the effects was quantified with Cohen's *f*. Significant effects were further characterized with post hoc contrasts, controlling the false‐discovery rate with Benjamini and Hochberg's procedure to address the multiple comparisons problem. Cohen's d was used to quantify the effect size in these pairwise contrasts. The analyses were conducted using R software (version 4.4.1).

## 3 Results

### 3.1 Startle Magnitude

To evaluate the possibility of group differences before the experimental manipulations, the raw (non‐standardized) startle magnitude on the five habituation trials was analyzed. A significant trial effect was found (*F*_(1,246)_ = 5.57, *p* = 0.017), but no group effect (*F*_(2,178)_ = 0.48, *p* = 0.61) nor interaction (*F*_(2,246)_ = 0.58, *p* = 0.55). This indicates that the groups did not differ in initial startle reactivity, nor in initial startle habituation.

The analysis of the startle magnitude of the target participants revealed a significant main effect of Time (*F*_(3,2319)_ = 56.18, *p* < 0.001, *f* = 0.27) and the predicted significant interaction Time × Group (*F*_(6,2319)_ = 2.64, *p* = 0.014, *f* = 0.08). Pairwise contrasts revealed that, during the speech phase, startle magnitude was markedly inhibited in participants performing the task alone compared to either those performing together with their partner (*t*_(938)_ = −2.398, *p*_FDR_ = 0.006, *d* = −0.19) or together with a stranger (*t*_(975)_ = −3.004, *p*_*FDR*_ = 0.005, *d* = −0.19).

The greater startle inhibition observed in the Alone group supports the idea that facing the TSST without social resources might impose a higher attentional load. By contrast, mere co‐presence, regardless of the level of relationship intimacy, may act as a stress‐buffer and render attentional investment more efficient.

**FIGURE 2:**
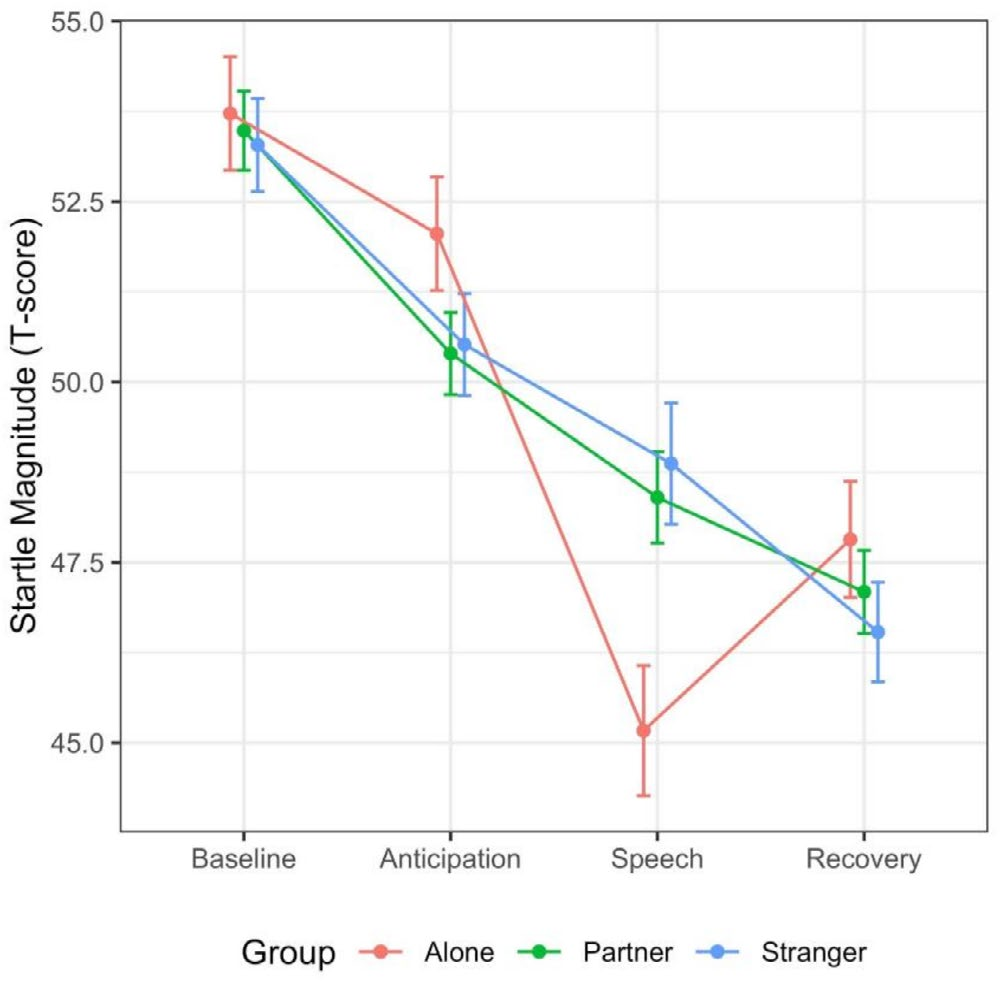
Standardized startle magnitude for the three groups in each phase of the TSST. Bars represent standard errors.

Additionally, contrasts revealed that participants in the Alone condition did not show startle habituation from baseline to anticipation (*t*_(2257)_ = 1.49, p_*FDR*_ = 0.13, *d* = 0.06), and from speech to recovery where they instead showed an increase in startle magnitude (*t*_(2280)_ = −2.19, p_*FDR*_ = 0.04, *d* = −0.09). Results are shown in Figure 2.

**FIGURE 3:**
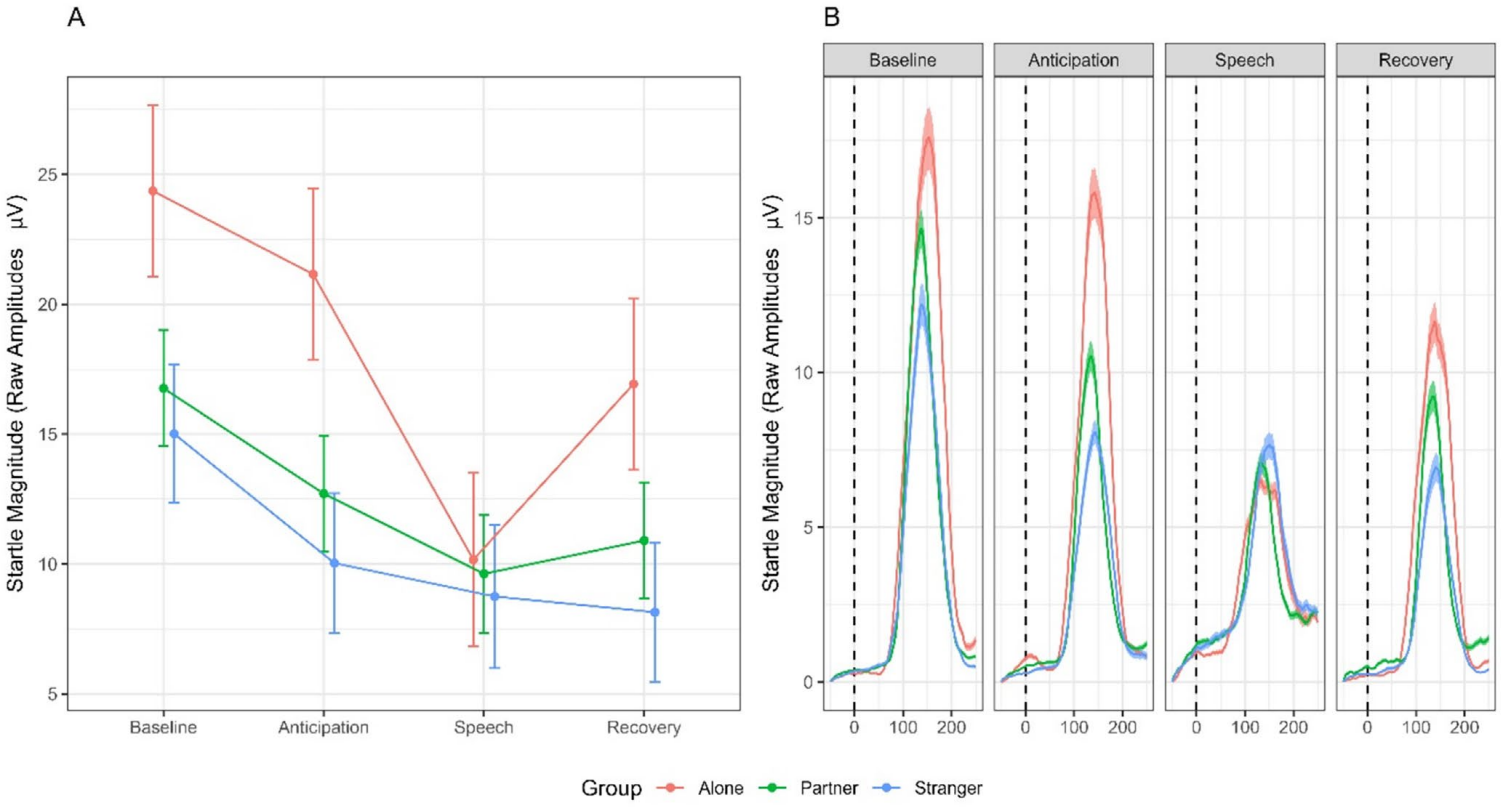
Panel A shows raw startle magnitude for the three groups in each phase of the TSST, with bars representing standard errors. Panel B shows the grand‐average waveform of the startle reflex for the three groups in each phase of the TSST, ribbons representing standard errors.

To assess the stability of these results, we also performed the same analysis using raw unstandardized data (Bradford et al. 2015). This analysis showed similar results, revealing a main effect of Time (*F*_(3,2319)_ = 61.42, *p* < 0.001, *f* = 0.29), and a significant Time × Group interaction (*F*_(3,2319)_ = 5.28, *p* < 0.001, *f* = 0.12) (Figure 3A). Pairwise comparisons (FDR‐corrected) revealed that the three groups were not different in baseline startle reactivity, but the Alone group showed a larger startle inhibition between the Anticipation and the Speech Phase (*t*_(2251)_ = 7.61, *p* < 0.001, *d* = 0.32), compared to the Partner (*t*_(2251)_ = 2.9, *p* = 0.1, *d* = 0.12) and the Stranger group (t_(2251)_ = 0.96, *p* = 0.9, *d* = 0.04). Furthermore, participants in the Alone group show an increase in startle magnitude in the transition from Speech to Recovery phase (*t*_(2251)_ = 4.63, *p* < 0.001, *d* = 0.2), which is absent in the Partner (*t*_(2251)_ = 1.23, *p* = 0.33, *d* = 0.05) and Stranger group (*t*_(2251)_ = 0.45, *p* = 0.75, *d* = 0.02).

This result confirms that the within‐group effect of the experimental manipulation is the same when considering raw or standardized startle magnitude. The most stressful phase of the TSST has the effect of eliciting a startle inhibition that is larger, relative to their general startle reactivity, in the Alone group compared to the Partner and Stranger groups.

### 3.2 Subjective Stress Reactivity

**FIGURE 4:**
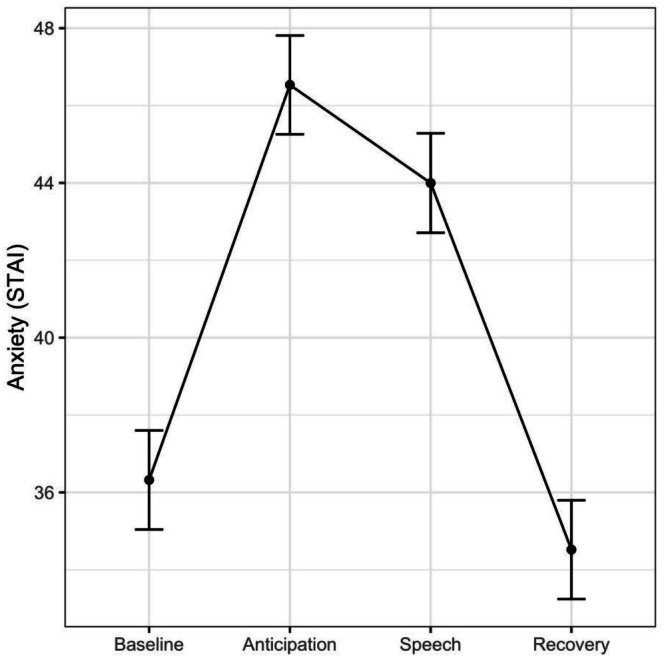
Self‐reported anxiety in each phase of the TSST. Bars represent standard errors. Data are collapsed across the three groups, due to the non‐significant Time × group interaction. See Supplementary Figure 1 for uncollapsed data visualization.

The analysis of self‐reported anxiety revealed only a significant main effect of Time (*F*_(3,201)_ = 43.05, *p* < 0.001, *f* = 0.8). Pairwise contrasts revealed the expected pattern of anxiety increase from the baseline phase to both the anticipation (*t*_(201)_ = −8.1, *p* < 0.001, *d* = −0.57) and speech phases (*t*_(201)_ = −6.07, *p* < 0.001, *d* = −0.43), followed by a decrease in anxiety from speech to recovery (*t*_(201)_ = 7.51, *p* < 0.001, *d* = 0.53). The analysis did not reveal a main effect of the Group (*F*_(2,68)_ = 0.33, *p* = 0.71, *f* = 0.1) nor its interaction with Time (*F*_(6,201)_ = 1.74, *p* = 0.11, *f* = 0.23). Results are shown in Figure 4.

The analysis of the ratings of perceived support from the presence of the *observer* revealed that participants in the Partner group felt more supported than participants in the Stranger group (*t*_(53)_ = 6.23, *p* < 0.001, M_Partner_ = 6.41, M_Stranger_ = 2.96), in line with the experimental manipulation. The analysis of the ratings of perceived discomfort induced by the presence of the *observer* revealed that participants in the Partner group felt similar levels of discomfort as participants in the Stranger group (*t*_(53)_ = 0.5, *p* = 0.61, M_Partner_ = 4.11, M_Stranger_ = 3.76).

## 4 Discussion

Social support is widely acknowledged as an important factor for efficient emotional regulation, especially when dealing with negative emotions such as emotional stress, and is crucial in shaping the degree of stress‐induced psychophysiological reactivity (Uchino 2009; Uchino et al. 2012). This is important especially in light of longitudinal findings showing how people who lack social support have an increased likelihood of developing health issues (e.g., cardiovascular disease), suggesting that in everyday life social support operates as a *buffer* against psychophysiological reactivity to stressors (Holt‐Lunstad et al. 2015; Wang et al. 2023). Thus, the buffering effect of social support prevents the accumulation of allostatic load and, eventually, the emergence of the known health‐damaging consequences of stress (Saxbe et al. 2020).

The majority of the research on this topic focused on characterizing this phenomenon in terms of changes in cardiovascular arousal and/or in terms of changes in neuroendocrine activity (i.e., cortisol). In this research, we wanted instead to focus on a different psychophysiological index, the startle reflex, which has been neglected so far, despite being a very sensitive measure of emotional as well as cognitive processing. Specifically, we were interested in (i) testing whether startle reactivity during a stressful task is affected by the availability of social resources, and (ii) testing if this effect is further shaped by the characteristics of the available social support, represented by either a romantic partner or stranger.

Our results revealed a rich pattern of effects. First, in line with our Hypothesis 1, we show that during a stressful task startle magnitude progressively decreases from the beginning to the climax of the task, with a marked inhibition in the acute stress phase. This is the first characterization of startle modulation during the TSST, complementing previous demonstrations of stress‐induced startle inhibition (De la Casa et al. 2016; Deuter et al. 2012). Here we observed a temporal profile of this modulation which confirms that the attentional demands of the TSST divert resources away from processing the auditory probes, and that startle inhibition is a valid marker of stress induction during this stress task. Nonetheless, it is important to acknowledge that the number of artifact‐free trials was lower in the Speech phase relative to the other phases, which could lead to a less precise estimation of the mean startle in this condition.

Second, in line with our Hypothesis 2, we found that the magnitude of stress‐induced startle inhibition depended on social context. In the analysis on standardized startle magnitudes (Figure 2), we show that during the acute stress phase, in the group of participants who performed the stressful task alone, startle is more inhibited compared to the other two groups of participants who faced the task with available social support. This shows that social presence buffered the attentional load imposed by the stressor and blunted startle inhibition. Additionally, the analysis of unstandardized data (Figure 3) reveals that this effect seems largely driven by the effect of social support in the anticipation of the threat. In this phase, participants in the Alone group have higher startle amplitude compared to the two social groups, and thus they experience speech‐induced inhibition to a larger extent. Finally, they also show an increase in startle in the transition from the Speech to the Recovery phase. This increase might be interpreted as a sign of stress not previously buffered by social support, able to induce a relative startle potentiation. Third, contrary to Hypothesis 3, no difference emerged between the Partner and Stranger conditions; the startle responses of the two social groups were comparable across all phases of the TSST.

The SBT provides a valuable framework for understanding the mechanisms subtending these effects. This theoretical model states that proximity to social resources is the *baseline* state in which our brain evolved to operate (Beckes and Sbarra 2022; Coan and Sbarra 2015). When dealing with the challenges and stress of daily life, social relationships, and more specifically, the availability of social support, decrease the predicted cost of the environment (Coan and Sbarra 2015). Availability of social support implicitly regulates how both cognitive and psychophysiological resources are invested for meeting environmental demands. In contrast, navigating the environment without social resources requires an additional metabolic effort for optimizing and regulating behavior.

Our pattern of results is consistent with this model, showing how available social resources buffer the stress and the cognitive load induced by the speech task. In our experimental paradigm, startle reactivity tracks this dynamic process by which proximity with social resources optimizes the physiological as well as cognitive regulation of behavior in an uncertain and potentially threatening environment. When participants start to anticipate the threat, access to social resources buffers anticipatory anxiety which results in lower startle reactivity. When this anticipation happens without access to social support, this leads to muted habituation and larger startle reactivity. Then, when facing the threat, the attentional demands of the task prompt a strong inhibition, larger in participants who are alone compared to those who are with another person who benefitted from social buffering in the anticipation. Finally, those who are alone show a relative stress‐induced increase in startle amplitude after the speech phase which is not present in the two social groups. This latter effect is interesting because it conceptually replicates a relatively recent finding showing how stress administered right before a fear‐learning paradigm has the effect of increasing resting startle reactivity and enhancing fear‐learning (Riggenbach et al. 2019). Here, we show that this stress‐induced startle enhancement might be buffered by the presence of social support.

Furthermore, our results suggest that this process is implicit. Indeed, our participants do not report any modulation in their levels of task‐induced anxiety as a function of available support nor its type. This is in line with evidence showing that the *stress‐buffering* effect of social support is robust at the physiological level, but does not necessarily extend to the psychological domain (Uchino et al. 2012). Here, we provide further support for this dissociation between the psychophysiological domain (i.e., startle reactivity) and the subjective domain (i.e., self‐reported anxiety). A possibility is that this dissociation stems from the different phenomena each measure taps. The startle reflex captures a rapid, largely implicit defensive response sampled every few seconds, whereas the STAI records consciously experienced anxiety only once per each phase. These differences in level (implicit vs. explicit) and temporal resolution make it unsurprising that the two channels do not align. Furthermore, it is worth highlighting that, in the context of research on the psychophysiology of stress‐buffering, patterns in which physiological processes are sensitive to support manipulation, but psychological processes are not, is the norm (Uchino et al. 2012). Indeed, weak concordance between physiological and subjective response to emotional manipulation has long been observed in psychophysiological research (Evers et al. 2014; Mauss et al. 2004, 2005; Quigley and Barrett 2014). These authors highlighted how relying on retrospective assessment of emotional state can lead to biased/imprecise measurement due to various mechanisms (e.g., cognitive reappraisal, defensive mechanisms, memory biases). On the other hand, continuous measurement of emotional experience is not always feasible without impending experimental manipulation. This is very much the case in the context of a highly ecological task such as the TSST. Furthermore, the personal relevance of the affective state induced by the TSST could have led to biased retrospective reports based on automatic emotional regulation strategies (e.g., suppression) and on social desirability, especially in the two social groups. Finally, to provide a balanced interpretation of the lack of group differences in task‐induced anxiety it is worth taking into account that the three groups were unbalanced in their size. Indeed, the lack of a significant effect may more simply reflect limited power to detect an interaction effect smaller than expected, rather than the absence of true differences in anxiety across the three groups. Future investigations should strive to improve on the limitations of our sample to resolve this issue.

Contrary to our Hypothesis 3 we did not find that the partner's presence provides more modulation than the stranger's. The main tenet of SBT is that social support enables the individual to outsource part of their threat vigilance to the other (Coan and Sbarra 2015). Thus, this process could be more efficient when the observer is a highly trusted and highly familiar person, such as the partner. In this study we found that having the partner present acts indeed as a source of support for participants, but nonetheless this feeling of support does not lead to a significant reduction in the levels of emotional stress nor produce a differential pattern in psychophysiological reactivity compared to having a stranger present. In order to explain this finding, we can speculate about the possibility that the relationship between social support and anxiety reduction is moderated by other external variables. Our study aligns with previous research that, while establishing a clear advantage of a social over an alone condition, reported mixed evidence with regards to the added benefit of the partner's presence compared to a stranger (Coan et al. 2006, 2017; Maffei et al. 2025). Several moderating variables have been suggested to explain this mixed evidence. Some are related to relationship‐specific features, such as relationship quality, relationship length, and perceived partner's responsivity in daily life (Ditzen and Heinrichs 2014; Saxbe et al. 2020; Uchino et al. 2012). Others are related to individual differences, such as social anxiety (Maffei et al. 2025) and attachment working models (Bourassa et al. 2019). In this study we did not measure these moderating variables, thus at present we cannot draw a strong conclusion about the implications of this null result in explaining whether partner's support is more efficient than support provided by a stranger.

Finally, another important aspect to consider is that the role of the partner as a source of social support changes in time, especially during the transition from late adolescence/emerging adulthood to early adulthood, when it becomes more central relative to other sources, such as family and friends (Galambos et al. 2018). Given the sample considered in this study, comprising only young (age < 25 years old) unmarried and non‐cohabiting couples, we must also consider the possibility that the lack of a partner‐specific effect might depend on the fact that for our participants the partner is not the most prominent buffering figure yet. Indeed, in our debriefing we found that participants in the Partner group felt similar levels of discomfort as the participants in the Stranger group induced by having someone else in the room with them. This can explain why in our research the partner's presence might be as effective as that of an unfamiliar person in buffering stress, and not more effective than a stranger.

Taken together, our results reveal that startle inhibition is a robust marker of stress‐induced affective and cognitive load, sensitive to the characteristics of the social environment in which the stressor occurs. In line with the prediction from the SBT, the analysis of the startle reflex during a stressful task shows how physical proximity with others automatically allows our neurocognitive systems to allocate resources more efficiently to cope with the demands of the task.

Nonetheless, it is important to acknowledge that our results should be interpreted considering also the limitations of this research. The main caveat of this research is that it is not easy to discern the effect of attentional modulation from the contribution of affective factors on the observed startle modulation. This is due to the characteristics of the task, on one hand, and the lack of a variable that is a relatively pure measure of attention, on the other. This caveat limits the possibility of establishing a causal involvement of attentional processes in the startle effects reported. Future studies should try to corroborate these findings, trying to disentangle the effect of social support on attention‐related startle inhibition from emotion‐related startle‐potentiation by employing more controlled experimental paradigms and convergent measures of attention, such as pupillometry (Strauch et al. 2022) or spontaneous eye‐blink rate (Maffei and Angrilli 2018). A potential approach could be employing the NPU‐threat test (Schmitz and Grillon 2012) comparing startle modulation induced by predictable/unpredictable threats across groups performing the task alone or with social support. Second, we recruited only female participants to participate in the role of the target. This is in line with previous research that showed how women benefit more than men from the process of stress buffering mediated by nonverbal presence (Ditzen and Heinrichs 2014). Furthermore, focusing only on female targets allowed us to minimize the potential confounding effects of gender differences that exist in affective and psychophysiological reactivity (Barnett et al. 2021; Maffei et al. 2015; Maffei and Angrilli 2019). Nonetheless, this choice limits the possibility of generalizing the observed pattern of effects to the male population as well as to same‐sex couples. A third limitation of our sample is its age, since it comprised only young dating couples, who had been together for a relatively short time and were not living together. This has likely limited our ability to detect specific effects related to the availability of the partner compared to the presence of a stranger and recommends testing for this effect in a more adult sample (i.e., older adults who are married/living together). Future studies should also aim at characterizing in detail the cortical and subcortical mechanisms that subtend this dynamic readjustment of resource allocation as a function of social support availability. Although the neural mechanisms involved in the generation of the startle reflex are known, with our study we cannot draw any conclusion about the cortical and/or subcortical network of regions involved in the observed startle modulation.

Finally, future work should also aim at characterizing how individual differences moderate the reported effects. Indeed, previous studies have shown that stress‐buffering effects are not uniform in their strength but are sensitive to both intra‐individual as well as inter‐individual (i.e., couple‐level) characteristics. Specifically, we believe that features related to social anxiety, emotion regulation abilities and preferred strategies (David et al. 2021; Del Popolo Cristaldi et al. 2022; Predatu et al. 2021), relationship quality and perceived support (Coan et al. 2017; Uchino 2009) are all important factors that, once modeled, could help further refine our understanding of how proximity with others helps us deal with stress.

## Author Contributions

**Antonio Maffei:** conceptualization, methodology, formal analysis, data curation, visualization, writing – original draft, supervision, project administration, funding acquisition. **Fiorella Del Popolo Cristaldi:** conceptualization, methodology, writing – review and editing. **Alessia Tecchio:** investigation, writing – review and editing. **Terry D. Blumenthal:** writing – review and editing, conceptualization. **Paola Sessa:** conceptualization, methodology, writing – review and editing.

## Conflicts of Interest

The authors declare no conflicts of interest.

## Supporting information


**Figure S1:** Self‐reported anxiety in each phase of the TSST and each experimental group.

## Data Availability

The data that support the findings of this study are openly available in OSF at https://osf.io/a52t7/?view_only=c6eea061153945f5a720520d81c270e1.
