# Supplementary figures and images for: Physical Proximity With Social Support Regulates Vigilance to Threat: Evidence From Startle Reactivity During Emotional Stress Induction
Source: Psychophysiology. 2026 Feb 10;63(2):e70259. doi: 10.1111/psyp.70259 (PMC12888949; doi:10.1111/psyp.70259)

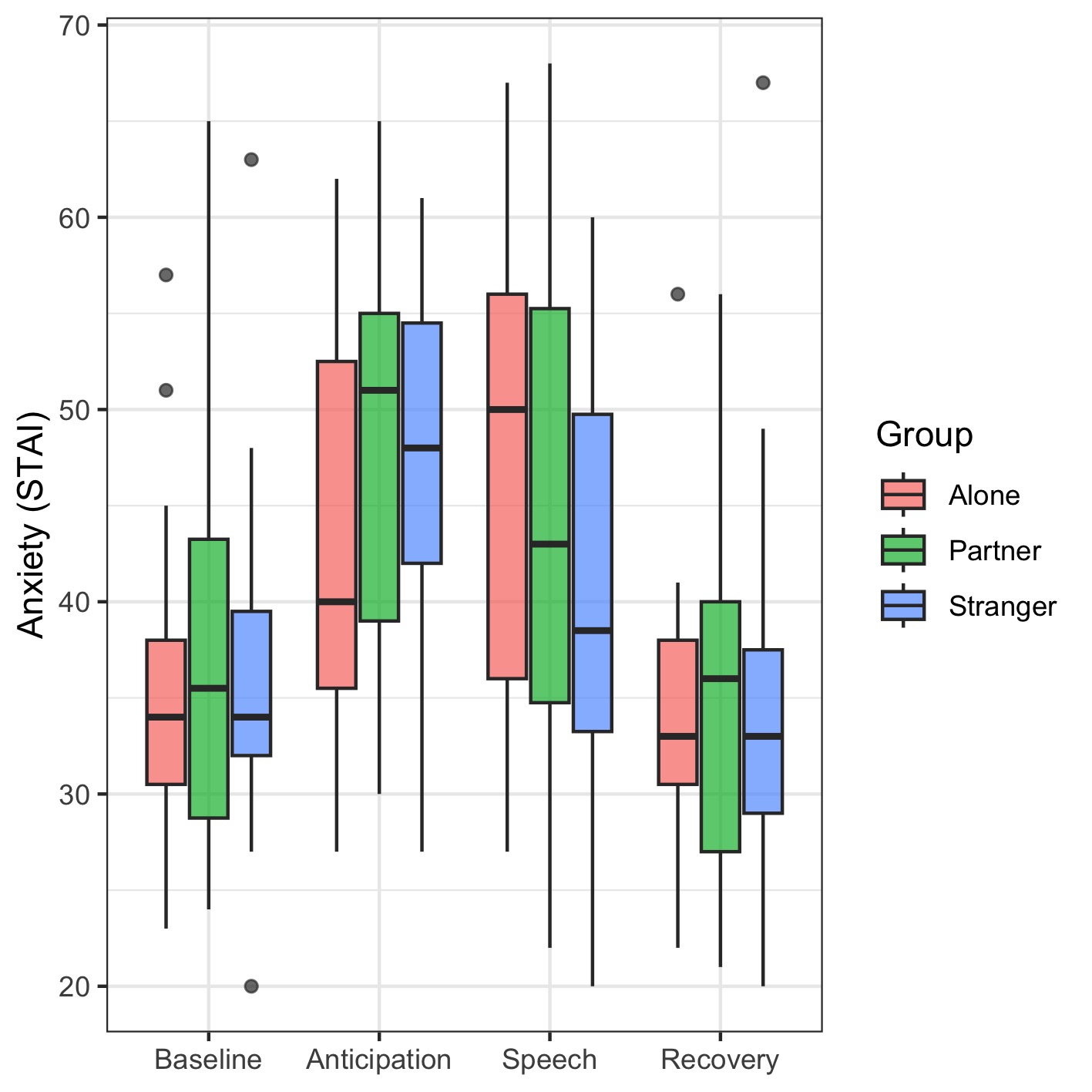

Supplement: Supplementary file 1 — Figure S1: Self‐reported anxiety in each phase of the TSST and each experimental group. [file PSYP-63-e70259-s001.jpeg]
